# Dual Agency: A Fresh Perspective to Identify Dilemma Mitigation Strategies – A Response to the Recent Commentaries

**DOI:** 10.34172/ijhpm.2022.7758

**Published:** 2022-11-20

**Authors:** Ruth Waitzberg, Wilm Quentin, Reinhard Busse, Dan Greenberg

**Affiliations:** ^1^Department of Health Care Management, Faculty of Economics & Management, Technical University Berlin, Berlin, Germany.; ^2^The Smokler Center for Health Policy Research, Myers-JDC-Brookdale Institute, Jerusalem, Israel.; ^3^European Observatory on Health Systems and Policies, Brussels, Belgium.; ^4^Department of Health Systems Management, School of Public Health, Faculty of Health Sciences, Ben-Gurion University of the Negev, Beer-Sheva, Israel.

 We were pleased to read the eight commentaries on our paper entitled “Dual agency in hospitals: how do managers and physicians reconcile clinical and economic considerations?,”^1^ and are happy to respond to them in this short correspondence. It was interesting to see how our article created a broad echo, and was relevant to various disciplines, ranging from medical ethics^2^ to sociology of professions,^3^ management of organizations,^4^ to name a few. Commentaries originate from several European countries (Spain, the Netherlands, Sweden, Germany, Norway, and the United Kingdom), Singapore and Australia (see Table). The variety of countries, disciplines and theories to which the commentators refer, reflect the strength of our interdisciplinary work, and the extent to which it was generalizable to other contexts and healthcare systems. Our paper combines theories and concepts from sociology of organizations, sociology of professions, and health economics, providing a rich and complex perspective of hospitals as organizations and the dynamics of their workers. While it is not always easy for authors from one discipline to incorporate theories and thinking from another, it opens opportunities to look at a phenomenon through new lenses.

**Table:** Overview of Commentaries by Authors, Affiliations and Discipline

**Author**	**Affiliation and Country**	**Discipline or Theory Mentioned**	**Main Comment**
Andersson^4^	University of Skövde, Sweden	Management of health organizations, institutional logics	“If hospital organizations are complex, we should let them be complex and instead learn to deal with this complexity, similar to what Waitzberg et al did.”
Bijlmakers^5^	Radboud University Medical Center, The Netherlands	Systems science, multi-criteria decision-making and evidence-informed deliberative processes	“Further work on reconciliation strategies, navigating diverging considerations and mitigating dilemmas may draw on the literature of multi-criteria decision-making and evidence-informed deliberative processes that are increasingly being used to optimise legitimate health benefit package design.”
Cascón-Pereira^3^	University Rovira i Virgili, Spain	Sociology of professions	“The instrumental use of the term dual agent to include ‘the other’ (the manager or the clinician) in a superlative and inclusive category can be considered a reframing strategy to solve inherent interprofessional conflicts and to implement more collaborative models in healthcare.”
Ewert^6^	Fulda University of Applied Sciences, Germany	Multi-faceted identity framework	“To theoretically capture professional hybridity in hospital management and beyond, applying a multi-faceted identity framework seems recommendable.”
Greener^7^	University of Strathclyde, UK	Social Policy	“Outlining the complexities of trying to balance clinical and managerial needs is important work, and being able to offer examples where compromises have been found that can be ‘win-win’ is a valuable contribution.”
Greve^8^	INSEAD, Singapore	Self-enhancement theory, management of organizations with multiple goals	“Through their emphasis on individual decision-making, they have given a view of how this conflict unfolds at the most micro level. For completeness, this should be coupled with a macro-level view.”
Lipworth^9^	Macquarie University, Australia	Medical values and role morality	“There is, however, one other way in which the dilemma between clinical and economic considerations could be addressed, and that is by physicians determining that they are responsible solely for patient care and leaving it to managers to address economic considerations.”
Sirris^2^	VID Specialized University, Norway	Medical ethics	“An important follow-up of the article is competency building among healthcare professionals and supporting the staff in the deliberation of clinical ethics dilemmas and policy development.”

 The strengths of the paper most highlighted by the commentaries related to the fresh view of the roles of hospital managers and physicians, and complexities of hospitals. The choice of the concept of the ‘dual agent,’ borrowed from health economics, to explore the decision-making of both managers and physicians avoided the dichotomy typically applied in sociology of professions. As clearly acknowledged by Cascón-Pereira, using the term ‘dual agents’ for all hospital workers highlights the common objectives instead of the dichotomies, and supports collaborative work towards mitigating conflicts and dilemmas.^3^ The unusual perspective of hospital managers and physicians as ‘dual agents,’ both attempting to reconcile between clinical and economic considerations added value to the narrative of dealing with dilemmas, as mentioned by Greener: “Outlining the complexities of trying to balance clinical and managerial needs is important work, and being able to offer examples where compromises have been found that can be ‘win-win’ is a valuable contribution. Too often doctors and managers are still presented as being inextricably in opposition to one another, when this does not always need to be the case.”^7^

 Among the criticism outlined in the commentaries, several authors dispute our assumptions. Lipworth points to the “lack of criticism to the values,” captured by noticing that no participant disputed the need to reconcile the clinical and economic considerations. Under his point of view, physicians and managers do have different priorities in their work and can only be committed to one priority at a time.^9^ Greener disputes our approach of learning from what works, and suggests scrutinizing what ‘failure situations’ could tell us. That could be explored by scrutinizing situations which managers and doctors attempt to avoid, or are not successful in resolving.^7^ While we prefer to learn from successes, learning from failures is also valuable to strengthen competencies of healthcare professionals and managers and to support them mitigate dilemmas.^2^ Ewert argues that the strategies of hospital professionals to align economic and clinical considerations are not durable, but rather a matter of serendipity. He assumes that activity-based payments divide hospital managers from physicians by its nature due to the economic incentives created and suggests reverting to global budgets. While global budgets create different economic incentives than activity-based payments, these can also result in perverse incentives to cut down service provision, avoid (high-cost) patients and reduce overall expenditures, eg, by using fewer staff or cheaper materials.^10,11^ In our view, global budgets and most payment methods in general, do not mitigate dilemmas of hospital managers and physicians – but that could be tested in future work.

## Calls for Future Work

 The concept of ‘dual agency’ could be further applied to other countries, as suggested by Sirris,^2^ and we further suggest using the concept also for analyzing perceptions of professionals in other settings such as outpatient specialist and primary care. In the context of primary care, general practitioners or nurses might be equally committed to their patients and the payer agency or insurer that contracts them. Different countries have different types of payers and payment methods for primary care, for example, health plans in Israel^12^ and Germany,^13^ local health authorities in Italy and Spain,^14,15^ municipalities in Norway,^16^ the National Health Service and clinical commissioning groups in England,^17^ community-based primary healthcare centers in Slovenia,^18^ just to name a few. Even among countries with the same type of payers, the type of contract with health professionals differs. For example, in Germany ambulatory care providers are often self-employed physicians, while in Israel, health plans directly employ many physicians. Employed professionals might be more committed to their employer than self-employed professionals are committed to the payer; physicians in more generous health systems might face fewer dilemmas between their commitment to the employer and the patients. But these are testable hypotheses. Understanding “dual agency” in different countries, settings and cultures, potentially sheds light on how these characteristics influence the types of dilemmas faced by health professionals, and the reconciliation mechanisms used to balance or align between different considerations. Similar to our comparison between Germany and Israel, identifying differences and similarities in dilemmas and coping mechanisms highlights the strategies that are generalizable to other contexts and those that are context specific.^7^ We invite researchers from other countries to explore the “dual agency” phenomenon in their in- and outpatient settings, to add, complement or dispute our findings.

 Commentators also suggest questions for future work. First, exploring other kinds of dilemmas beyond those that misalign economic and clinical considerations, which we acknowledge, and hope to pick up in future work.^1,5^ We might further identify other dilemmas, to create a richer picture of the dynamics of hospital and other health workers within their complex work environment. During the coronavirus disease 2019 (COVID-19) pandemic, new dilemmas have surfaced, such as the commitment to COVID-19 and non-COVID-19 patients and who to prioritize, the commitment to vaccinated and unvaccinated patients, and the commitment to handling health shocks along with “routine” care. The pandemic might have exacerbated the already existing dilemmas, as it aggravated the challenges in place before, for example, shortage of health workers, funding or infrastructure. We could explore the effects of the COVID-19 pandemic through the “dual agency” lenses and learn whether and how dilemmas were created or exacerbated, and which coping mechanisms were applied.

 In summary, it seems that our paper has made an important contribution to the literature by showing that the concept of “dual agency” can be helpful for research that aims to unveil how professionals in complex organizations with multiple objectives cope with dilemmas and conflicts. We hope to see this being applied more broadly by researchers in other contexts in the field of health policy and management.

## Ethical issues

 Not applicable.

## Competing interests

 Authors declare that they have no competing interests.

## Authors’ contributions

 All authors drafted the work, revised it critically, and approved of the version to be published. All authors agree to be accountable for all aspects of the work.
